# Are Unique Regional Factors the Missing Link in India’s COVID-19-Associated Mucormycosis Crisis?

**DOI:** 10.1128/mbio.00473-22

**Published:** 2022-03-31

**Authors:** Jessy Skaria, Teny M. John, Shibu Varkey, Dimitrios P. Kontoyiannis

**Affiliations:** a Independent Researcher, Houston, Texas, USA; b Department of Infectious Diseases, Infection Control and Employee Health, The University of Texas MD Anderson Cancer Center, Houston, Texas, USA; c Maxivision Eye Hospitals, Trichy, Tamil Nadu, India; CDC

**Keywords:** COVID-19-associated mucormycosis (CAM), cow excrement, COVID-19, cow excreta, missing link, mucormycosis

## Abstract

The exact cause of the disproportionate increase in COVID-19-associated mucormycosis (CAM) cases in India remains unknown. Most researchers consider the major cause of India’s CAM epidemic to be the conjunction of the COVID-19 pandemic and associated corticosteroid treatment with the enormous number of Indians with diabetes mellitus (DM). However, excess CAM cases were not seen to the same extent in the Western world, where diabetes is prevalent and corticosteroids are also used extensively for COVID-19 treatment. Herein, we hypothesize that previously overlooked environmental factors specific to India were important contributors to the country’s CAM epidemic. Specifically, we propose that the spread of fungal spores, mainly through fumes generated from the burning of Mucorales-rich biomass, like cow dung and crop stubble, caused extensive environmental exposure in the context of a large population of highly vulnerable patients with DM and COVID-19. Testing this hypothesis with epidemiologic studies, phylogenetic analyses, and strategic environmental sampling may have implications for preventing future epidemics.

## OPINION/HYPOTHESIS

**FIG 1:**
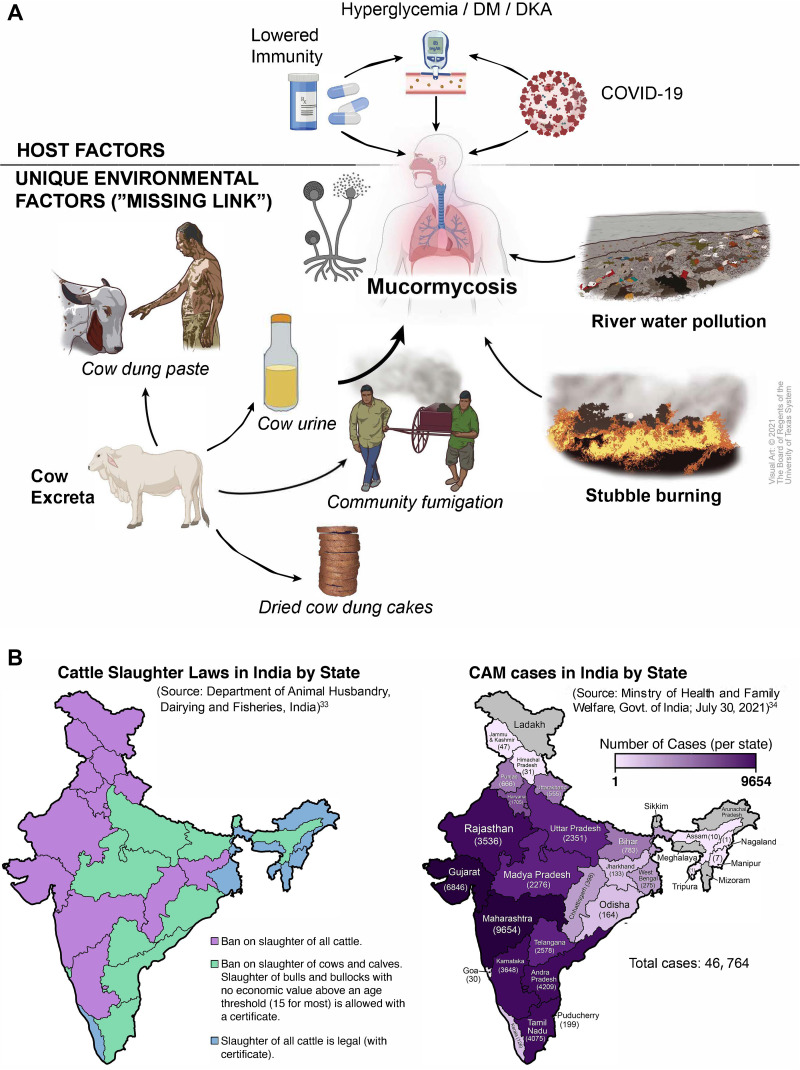
(a) The intersection of known host factors with unique environmental factors specific to India potentially accounted for the CAM epidemic. (b) Comparative maps of India showing the relative clustering of CAM cases in states that ban cow slaughter. (Data were obtained from the Government of India’s Department of Animal Husbandry, Dairying, and Fisheries [33] and Ministry of Health and Family Welfare [34]).

Compared with other countries, India has always had a disproportionate mucormycosis caseload (1), but why COVID-19-associated mucormycosis (CAM) was seen in such large numbers and in so short a time span in only India remains somewhat unclear. The number of CAM cases in India increased sharply in late April 2021 (2), in conjunction with the second wave of the COVID-19 pandemic, and the government declared CAM an epidemic on 20 May. The number of cases in the country exceeded 41,000 by mid-July (3) and plateaued in August; currently, the country’s average mucormycosis caseload is nearly the same as it was before the COVID-19 pandemic, albeit still significantly higher than the global average. In contrast, CAM remains relatively uncommon in other nations despite those countries’ high prevalence of diabetes mellitus (DM) and widespread use of corticosteroids for COVID-19 treatment, the factors most commonly cited as causes of the Indian epidemic (4–7). For instance, the prevalence of DM among adults is 13.6% in the United States, 13.0% in China, 10% in Germany, 8.6% in France, and 6.8% in the Netherlands but 8.3% in India (8). Although it does not have the highest prevalence of DM, India has 74.2 million adults with DM and another 39.4 million with undiagnosed DM and is, thus, second only to China in terms of people living with DM (8). Despite their unproven efficacy in non-hypoxic patients with COVID-19, corticosteroids are frequently used to treat patients with mild or moderate COVID-19 in other parts of the world as well (9, 10). This raises the question of whether excessive environmental exposure to Mucorales spores is the unacknowledged factor in India’s CAM epidemic. One recent multicenter study from India showed that the Mucorales burden in the outdoor hospital environments in India is as high as 51.8% (11). Herein, we posit that important but overlooked cultural, religious, and social factors specific to India might be responsible for the excessive burden of fungal spores, including Mucorales spores, in the Indian environment. Specifically, we hypothesize that Mucorales-rich cow excrement, given its use in multiple Indian rituals and practices, especially during the pandemic, probably played a key role in India’s CAM epidemic. We also posit that the dispersal of the fungal spores most likely occurs through fumes generated from the burning of Mucorales-rich biomass, such as cow dung and crop stubble. We believe that this dispersal likely resulted in a heavy load of Mucorales in and around vulnerable immunocompromised and diabetic COVID-19 patients, thus helping drive India’s CAM epidemic (Fig. 1a).

Cow excrement is rich in Mucorales, which are coprophilous (dung-loving) fungi with a particular affinity for herbivore dung (12–15). Twenty-four different taxa of Mucorales, including *Mucor*, *Rhizopus*, and *Cunninghamella*, have been identified in herbivore dung (15). India, with more than 300 million cattle, ranks highest in the global cattle inventory. Incorporating cow excreta into daily life is a longstanding tradition in India (16, 17), significantly more so than in the rest of the world. Many Indians consume products that contain cow excreta; for example, Panchagavya, which is ingested as an Ayurvedic medicine, contains milk, ghee, and curd but also cow dung and urine (18). In addition, common rituals in parts of India include applying cow dung on bodies, drinking cow urine, and burning and inhaling cow dung fumes as a form of ritual purification during festivals, prayers, or cremations. Herbivore dung is also used as cooking fuel in rural India. Even before the COVID-19 pandemic, several sociocultural activities, including the burning of cow dung, and agricultural practices, like stubble burning, were possibly correlated with the high environmental loads of Mucorales, as biomass fires can disperse viable fungal spores (19, 20). The post-harvest practice of stubble burning, which potentially increases fungal spore dispersion both from vegetation and manure-rich agricultural soil, could also explain the prepandemic seasonal variations in mucormycosis, which had a higher incidence in the fall and early winter (21). People exposed to the above practices are hence likely to have their nasal passages more densely colonized by Mucorales spores, thereby increasing the chances of at-risk hosts developing mucormycosis.

The personal and communal use of cow excreta among Indians increased substantially during the COVID-19 pandemic. Encouraged by political and religious rhetoric, many Indians regularly used copious amounts of cow dung and urine in the hopes of preventing and treating COVID-19 (22, 23). Ritual mass fumigations (*havans*) with dense smoke generated by smoldering dried cakes of cow dung and ghee—potentially facilitating large airborne spore dispersal—were claimed to have virucidal effects (24) and were conducted in the open in multiple urban and rural localities throughout India (25–27). Crematoriums, unable to keep pace with the unprecedented death rate, substituted cow dung for wood as fuel (28). The use of Gomutra Arka, or cow urine distillate, was claimed to be a successful COVID-19 treatment (29). These practices possibly resulted in unusually large fungal loads that markedly increased the risk of invasive fungal infections among COVID-19-susceptible Indians. Cow dung, which harbors multiple fungal species, could also explain the instances of mixed fungal infections, such as concomitant mucormycosis and aspergillosis, which were reported in as many as one-third of CAM patients in India (13, 30, 31).

The practices described above are not uniformly observed across India, however. The veneration of the cow and use of cow excreta-containing products are more common in states with stricter adherence to Hindu religious practices (32). These states typically also have laws banning cow slaughter (33). Remarkably, CAM cases were substantially more common in states that ban cow slaughter (34) and whose residents are more likely to use cow excreta (Fig. 1b). In fact, several incidents of mass rituals and fumigations using cow dung in these states (25–27, 35) were followed by media reports of clusters of mucormycosis in the same areas (36–39). In contrast, the incidence of CAM was much smaller in states where cow slaughter is allowed and beef is consumed and residents are less likely to use cow excreta. For example, the state of Kerala, which has the most liberal cow slaughter laws and the highest prevalence of DM in India (40), led the nation in the number of COVID-19 cases (41) but had one of the smallest caseloads of CAM (34). The differences in CAM incidence between adjacent Indian states (such as that between Kerala and both Tamil Nadu and Karnataka, which had high caseloads of CAM) and between India and neighboring countries with a similar climate does not support climatologic factors as drivers of mucormycosis.

That the CAM epidemic occurred shortly after major Hindu religious festivals is also unlikely to be a coincidence; however, the crowding at such festivals could have contributed to increased cases of COVID-19 and consequently increased numbers of vulnerable hosts. In 2021, the Festival of Holi was celebrated in March, and the Kumbh Mela was held in April, preceding the second COVID-19 wave (42, 43). These festivals typically involve the increased use and burning of cow dung, and devotees also bathe in holy river waters. Mucorales are known to exist in unpolluted surface waters (44) and probably have an even higher incidence in polluted waters. The practice of burying or burning corpses at the embankments of holy rivers in India, which escalated during the peak of the pandemic (45), could also increase the presence of microbial pathogens in those waters. Notably, cities located alongside rivers had higher incidences of mucormycosis (46). That the mucormycosis rate increases following natural disasters like tsunamis (47) also points to a potential waterborne route of entry. This has added importance in the Indian context, since water from the Ganga has been touted as a cure for COVID-19 (48).

Although it does not exclude the possible contribution of cow dung use to CAM cases, the use of untested remedies, such as Coronil or 2-deoxyglucose, might have exacerbated the metabolic and/or immune dysregulation of COVID-19 patients with DM and, in the background of excessive environmental exposure to Mucorales spores, contributed to the country’s high CAM incidence (49).

In determining likely causes of excess CAM cases, one could draw global parallels between the use of cow dung in India and the use of other types of animal dung in other countries. For example, Anbarnesa smoke, a traditional medicine derived from the burning of donkey dung, was used extensively during the COVID-19 pandemic in Iran (50), a country that has also experienced an unusual increase in CAM cases (51).

India’s experience with neonatal tetanus (NNT) could draw some interesting parallels with the CAM epidemic in that country, even though NNT and mucormycosis have different pathophysiologies. In the 1980s, NNT, already eliminated in the developed world, accounted for more than 200,000 neonatal deaths annually in India (52). Questions as to how India could uniquely have such a high prevalence of NNT were raised. Collaborative studies by the World Health Organization and Indian government showed that the predominant causes for India’s asymmetric prevalence were low rates of antenatal vaccination and high rates of home births under unhygienic conditions (53). In relation to the latter, application of cow dung on the newborn’s umbilical cord stump was epidemiologically identified as an important risk factor (54). As is the case with Mucorales (15), animal dung also contains abundant tetanus spores (55). Although a variety of strategies (increased coverage of maternal tetanus immunization, promotion of institutional deliveries through cash incentives, promotion of safe and hygienic delivery and umbilical cord practices) led to the eradication of NNT in India (56), both conditions bring into focus the potential role of spore-forming ubiquitous microbes of cow dung used in the sociocultural context and disproportionate infectious disease burden in India. If our hypothesis proves to be accurate, lessons learned from the management of NNT may be valuable in controlling mucormycosis as well.

Verifying our hypothesis could have important implications for the prevention of CAM, and mucormycosis in general, in India and worldwide. Case-control studies assessing differences in demographics and Mucorales spore exposure between patients with CAM and patients with COVID-19 without mucormycosis in at least three different areas with disparate climatological characteristics would show whether disproportionate environmental spore exposure is a potential risk factor for CAM. In addition, phylogenetic analyses would show whether Mucorales isolates from CAM patients are identical to those from herbivore excreta from the same area. Finally, pyroaerobiological analyses of biomass samples and associated bioaerosols would show whether Mucorales spores can aerosolize and remain viable after combustion. Further research in these directions would hopefully establish the role of herbivore dung in the causation of CAM and lay the foundation for initiating measures to reduce the incidence of the disease.
